# Manganese Is a Strong Specific Activator of the RNA Synthetic Activity of Human Polη

**DOI:** 10.3390/ijms23010230

**Published:** 2021-12-26

**Authors:** Eva Balint, Ildiko Unk

**Affiliations:** Biological Research Centre, Institute of Genetics, Eotvos Loránd Research Network, H-6726 Szeged, Hungary; balint.eva@brc.hu

**Keywords:** human polymerase η, RNA extension, manganese, translesion synthesis, enzyme kinetics

## Abstract

DNA polymerase η (Polη) is a translesion synthesis polymerase that can bypass different DNA lesions with varying efficiency and fidelity. Its most well-known function is the error-free bypass of ultraviolet light-induced cyclobutane pyrimidine dimers. The lack of this unique ability in humans leads to the development of a cancer-predisposing disease, the variant form of *xeroderma pigmentosum*. Human Polη can insert rNTPs during DNA synthesis, though with much lower efficiency than dNTPs, and it can even extend an RNA chain with ribonucleotides. We have previously shown that Mn^2+^ is a specific activator of the RNA synthetic activity of yeast Polη that increases the efficiency of the reaction by several thousand-fold over Mg^2+^. In this study, our goal was to investigate the metal cofactor dependence of RNA synthesis by human Polη. We found that out of the investigated metal cations, only Mn^2+^ supported robust RNA synthesis. Steady state kinetic analysis showed that Mn^2+^ activated the reaction a thousand-fold compared to Mg^2+^, even during DNA damage bypass opposite 8-oxoG and TT dimer. Our results revealed a two order of magnitude higher affinity of human Polη towards ribonucleotides in the presence of Mn^2+^ compared to Mg^2+^. It is noteworthy that activation occurred without lowering the base selectivity of the enzyme on undamaged templates, whereas the fidelity decreased across a TT dimer. In summary, our data strongly suggest that, like with its yeast homolog, Mn^2+^ is the proper metal cofactor of hPolη during RNA chain extension, and selective metal cofactor utilization contributes to switching between its DNA and RNA synthetic activities.

## 1. Introduction

The intra- and extracellular environment produce agents that can be harmful to cells inheriting DNA molecules via introducing strand breaks or chemical linkage between adjacent bases or modifying the sugar or the base components of their DNA. Alteration or damage in DNA can potentially stall replication due to the high selectivity of the replicative DNA polymerase. Stalled replication can lead to DNA strand breaks, genomic rearrangements, and finally to cell death. To circumvent such fatal consequences, cells have evolved different DNA damage tolerance mechanisms that can ensure the continuity of replication without removing the damage. One of those mechanisms is direct synthesis across the damage site by translesion synthesis (TLS) DNA polymerases. The Y family of polymerases consists of TLS DNA polymerases capable of synthesizing across DNA damage with relatively high efficiency [1,2]. They can do so because their active center is more spacious and less selective than classical DNA polymerases enabling them to accommodate modified nucleosides. However, their low selectivity renders TLS polymerases error-prone, often inserting incorrect nucleotides opposite DNA lesions. As a result, TLS is often mutagenic and contributes to cancer development [3,4]. The complexity of the roles of TLS polymerases is highlighted by the fact that their inactivity or absence can also advance cancer formation [5,6,7,8]. Human DNA polymerase η (hPolη) is a TLS polymerase with the unique ability to efficiently and without error bypass cyclobutane pyrimidine dimers (CPDs), one of the most frequent UV-induced DNA lesions [9,10]. Inactivity of hPolη leads to the development of xeroderma pigmentosum variant (XP-V) form that predisposes ultraviolet light (UV)-exposed individuals to cancer due to error-prone bypass of CPDs by other TLS polymerases. The other cognate DNA lesion of hPolη that it can bypass efficiently and largely without error is 7,8-dihydro-8-oxo-2-deoxyguanosine triphosphate (8-oxoG), one of the most prevalent oxidative lesions, but hPolη can also bypass a wide range of DNA lesions with varying fidelity [11,12,13,14,15,16].

hPolη, like most other DNA polymerases, can misinsert ribonucleosides (rNMPs) during DNA synthesis, contributing to the accumulation of rNMPs in the genome, although with a thousand-fold lower efficiency than dNMPs [17,18,19,20]. Embedded rNMPs represent probably the most abundant DNA lesions in the genome of eukaryotic cells but are efficiently removed by RNase H2-dependent ribonucleotide excision repair [21,22,23]. The role and consequences of rNMPs in DNA are still debated, but their detrimental effect could contribute to the development of Aicardi–Goutières Syndrome and Systemic Lupus Erythematosus, both linked to mutated RNase H2 [24,25]. hPolη can insert rNMPs opposite DNA lesions as well [18,20]. Experiments using XP-V cell extracts indicated that hPolη was the key source of rCMP incorporation across cisplatin intrastrand guanine crosslinks [20]. In addition, hPolη can extend RNA primers with rNTPs and shows a reverse transcriptase activity as it is able to synthesize DNA using RNA templates even opposite an 8-oxo-rG lesion, with comparable efficiency to using DNA templates [26,27]. Based on the latter two activities, it was suggested that hPolη participates in DNA damage bypass during RNA primer synthesis on the lagging strand during Okazaki fragment replication, and in double-strand break repair where it seals the gap using a transcript RNA as template [26].

Our previous studies with the yeast *Saccharomyces cerevisiae* Polη (yPolη) revealed its unexpected involvement in transcription [28]. We demonstrated that the lack of yPolη caused defects in transcription, particularly in transcription elongation in yeast cells. Moreover, we showed that yPolη could extend RNA primers using rNTPs on both undamaged templates and opposite TT dimer and 8-oxoG DNA lesions. Importantly, we found that the weak efficiency of RNA synthesis observed in the presence of the metal cofactor Mg^2+^ was enhanced several thousand-fold using Mn^2+^ without lowering the base selectivity of the enzyme on undamaged templates, as well as during DNA lesion bypass [29]. Other metal cations, however, did not support RNA synthesis by yPolη. Together, these results strongly suggested that Mn^2+^ was a specific activator of the RNA synthetic and translesion RNA synthetic activities of yPolη, and the enzyme utilized different metal cations to promote its DNA or RNA synthetic activities.

In this study, we investigated whether the weak RNA synthetic activity of hPolη could be improved by applying different metal cofactors in the reactions. Our results show that Mn^2+^ greatly enhances RNA extension by hPol compared with Mg^2+^ without decreasing the base selectivity of the polymerase. Activity increase could be observed opposite TT dimer and 8-oxoG DNA lesions as well. Our results suggest that, as with its yeast counterpart, Mn^2+^ is a specific activator of the RNA synthetic and translesion RNA synthetic activities of hPolη. We discuss the possible biological significance of the greatly improved RNA synthetic activity.

## 2. Results

### 2.1. Metal Cation Dependency of the RNA Extension by hPolη

**Figure 1:**
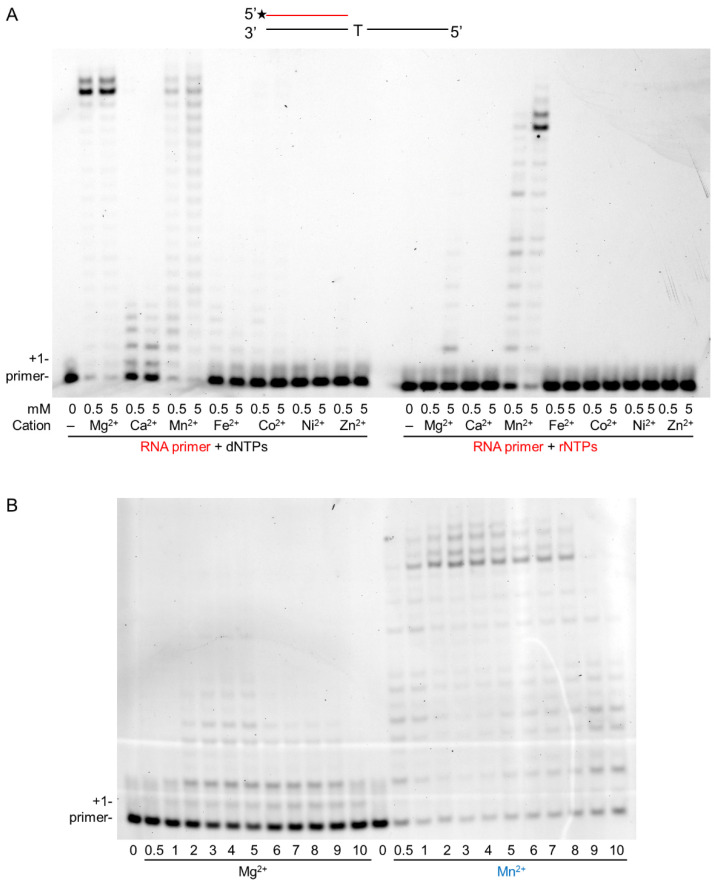
Manganese activates the RNA synthetic activity of hPolη. (**A**) Primer extension reactions were performed applying two different concentrations of the indicated divalent cations. The structure of the primer/template used in the experiments is shown on the top. The black line depicts a DNA strand, and the red line is an RNA strand. The asterisk indicates a fluorescent label. The first templating nucleotide is shown. Reactions contained 30 nM Polη, 20 nM primer/template, and either 50 μM of dNTPs (left panel) or 1 mM of rNTPs (right panel). The positions of the primer and its one nucleotide extension are indicated. (**B**) Mg^2+^ and Mn^2+^ concentration-dependent RNA extension. Reactions were performed for 10 min with 20 nM hPolη in the presence of 20 nM RNA/DNA and 1 mM rNTPs. The concentrations of Mn^2+^ and Mg^2+^ are indicated below each lane.

The RNA extension ability of hPolη was tested using seven different metal cations at low, 0.5 mM, and at high, 5 mM, concentrations in in vitro primer extension reactions containing a DNA template, an RNA primer, and all four nucleotides at equal concentrations. The gel picture in Figure 1A shows that all tested cations supported synthesis to varying degrees using dNTPs, but Mg^2+^ and Mn^2+^ were the most effective, resulting in the extension of almost all of the added RNA primers. In contrast, Ni^2+^ and Zn^2+^ were the least effective. Importantly, using rNTPs, only Mn^2+^ catalyzed robust synthesis, even at low concentration, whereas Mg^2+^ was a weak activator but only at high concentration. The other metal cations did not support the reaction. These results suggested Mn^2+^ as the metal cofactor for hPolη during RNA synthesis and Mg^2+^ as a weak substitution. Before characterizing RNA synthesis using the two cations, first, we determined the optimal concentration of Mg^2+^ and Mn^2+^ in the reactions. As Figure 1B shows, both ions catalyzed RNA extension at a wide range of concentrations, with the highest activities detected using 3–5 mM of either metal ions, therefore we applied 4 mM of each in the primer extension assays during this study.

### 2.2. Manganese Is a Specific Activator of hPolη during RNA Extension

**Figure 2:**
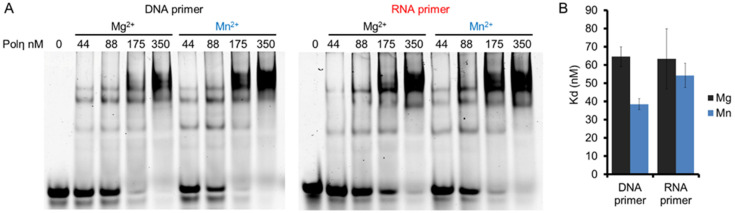
The binding affinity of hPolη to DNA and RNA primers in the presence of Mg^2+^ or Mn^2+^. (**A**) DNA/DNA and RNA/DNA primer/templates (20 nM) were incubated with 0 to 350 nM hPolη as indicated, in the presence of 4 mM Mg^2+^ or Mn^2+^. Complexes were resolved on a 4% non-denaturing polyacrylamide gel. (**B**) Quantitation of binding affinities. 10 nM of DNA/DNA or RNA/DNA was incubated with hPolη (from 22 to 175 nM in 11 increments). The standard deviation (SD) for each substrate is indicated.

The effects of Mg^2+^ and Mn^2+^ on RNA synthesis by hPolη were investigated by first checking whether the two metal ions conferred distinct affinities to hPolη toward the primer/template. We applied DNA/DNA and RNA/DNA primer/templates in electrophoretic mobility shift assays (EMSA), together with increasing concentrations of hPolη, and with Mg^2+^ or Mn^2+^ in the reactions. The results of these experiments demonstrated that hPolη could bind the primer/templates in the presence of both Mg^2+^ and Mn^2+^, but the binding was slightly stronger with Mn^2+^ (Figure 2A,B). Fitting the quantitated data to the Hill equation revealed the Hill coefficients, which were 2.0 ± 0.2, 1.8 ± 0.2 1.9 ± 0.5, and 1.8 ± 0.3 for DNA primer with Mg^2+^, DNA primer with Mn^2+^, RNA primer with Mg^2+^, and RNA primer with Mn^2+^, respectively, indicating that 2 hPolη molecules bound to a single substrate molecule in each case. In reactions with Mg^2+^, hPolη bound the DNA/DNA and RNA/DNA primer/template with similar affinities, with dissociation constants (K_d_) ~60 nM. Mn^2+^ conferred almost the same affinity toward RNA/DNA (K_d_ ~55 nM) and a somewhat stronger affinity toward DNA/DNA (~40 nM), indicating that oligonucleotide binding did not contribute to the RNA extension enhancement observed with Mn^2+^.

**Figure 3:**
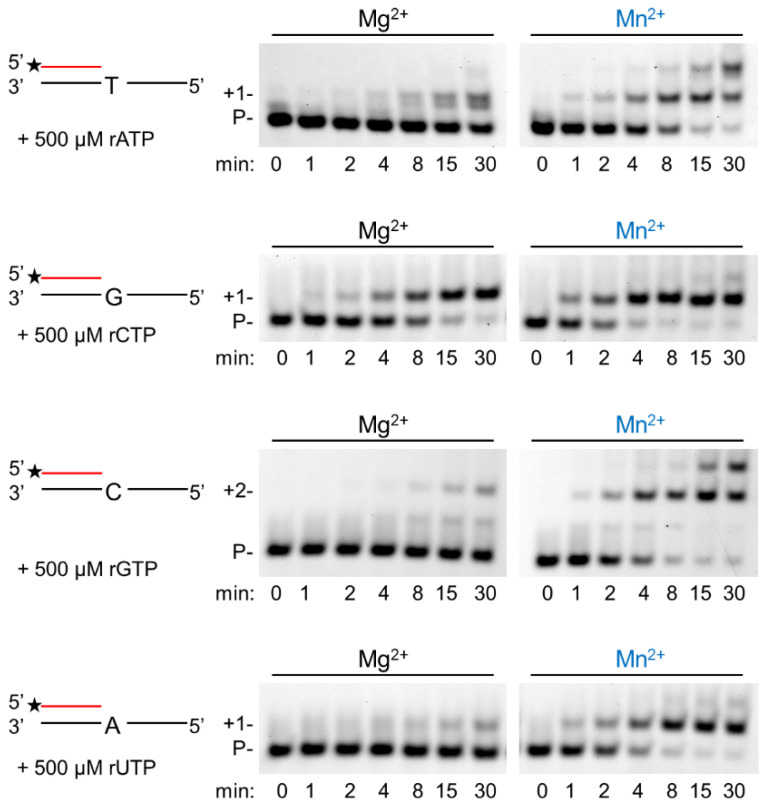
Manganese enhances the velocity of the polymerization reaction. Primer extension reactions were performed with 5 nM hPolη, 20 nM RNA/DNA, 500 μM of individual rNTPs, and 4 mM Mg^2+^ or Mn^2+^ for the indicated times. Labels are the same as in Figure 1.

**Figure 4:**
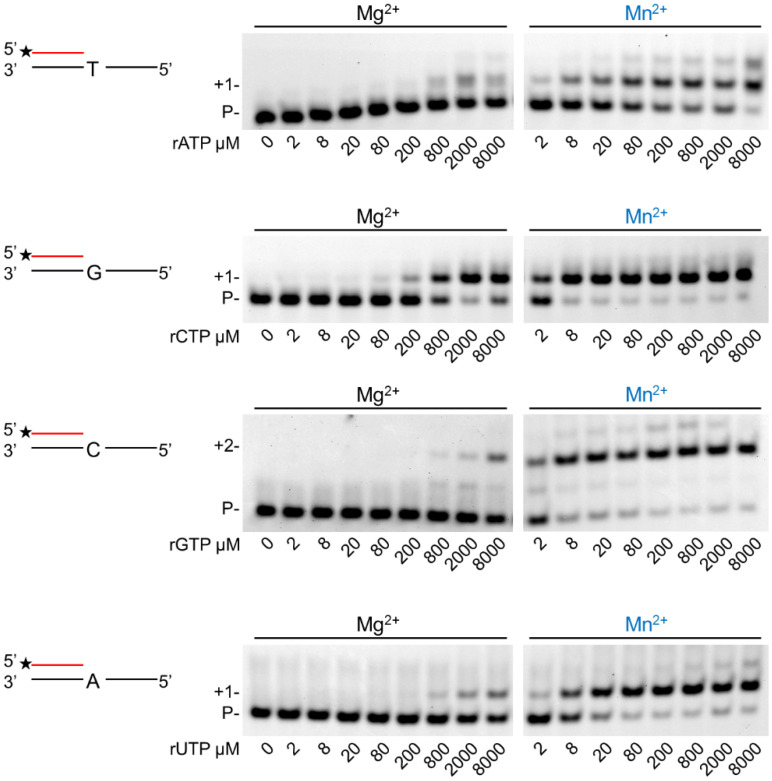
Manganese enhances hPolη’s affinity to ribonucleotides. Reactions were performed using 1 nM enzyme, 20 nM RNA/DNA, 4 mM Mg^2+^ or Mn^2+^, and various concentrations of individual rNTPs as indicated. Reaction time was 45 min. Labels are the same as in Figure 1.

In the next step, we assessed the velocity of RNA extension using the two cations. hPolη was incubated with 500 µM of each rNTPs separately for increasing times. As Figure 3 shows, RNA extension was faster with Mn^2+^ than with Mg^2+^, although the difference was only a few folds. Next, we examined the effect of the metal cofactors on the affinity of the enzyme to ribonucleotides. Primer extension was carried out with increasing concentrations of the single incoming nucleotide. The representative gel pictures in Figure 4 show that hPolη had a much higher affinity to rNTPs when Mn^2+^ was applied as the metal cofactor. To get a more unequivocal picture, we determined the kinetic parameters of the extension by steady-state kinetic analysis. These experiments confirmed that the catalytic constants (k_cat_) indicating the velocity of rNMP insertion were almost the same with either Mg^2+^ or Mn^2+^, since the highest increase, observed during UMP insertion, was less than three-fold, in good agreement with the data presented above (Table 1). In contrast, the Michaelis–Menten constants (K_m_) showing the affinity of the enzyme to the substrate were several hundred-fold lower in the presence of Mn^2+^, reflecting a higher affinity of hPolη to rNTPs. Together these changes resulted in a 428-fold efficiency increase with the correct rCTP, a 774-fold in the case of the correct UTP, and a 1260-fold increase with the correct incoming rGTP. We note, however, that we could not compare rAMP insertion in the presence of the two metals, because Mg^2+^ facilitated the insertion of a dATP contamination in rATP, as indicated by the appearance of a faster mobility band below the position of +1 extension (Figure 3 and Figure 4). 

### 2.3. Fidelity of RNA Extension Using Manganese

**Figure 5:**
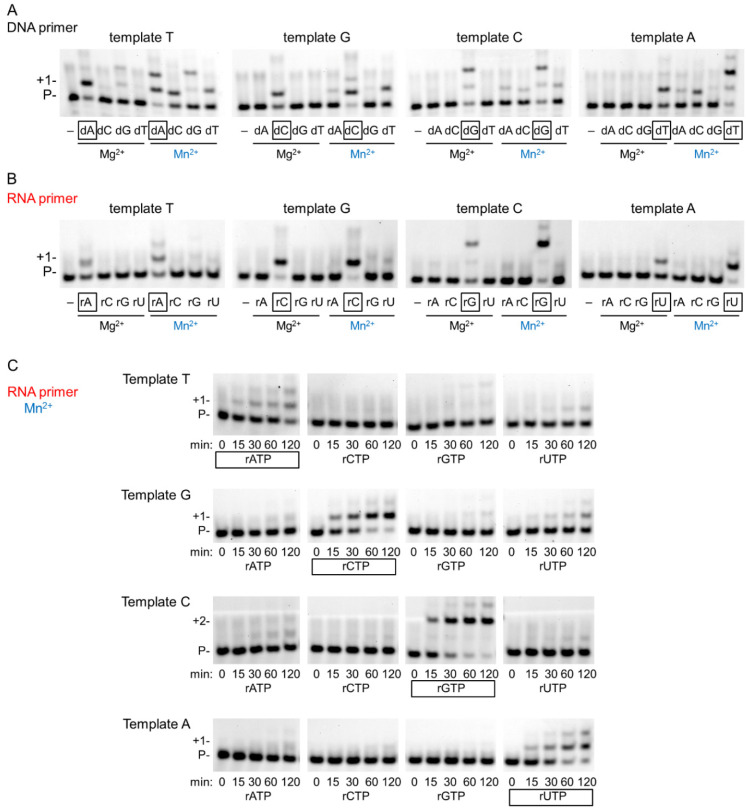
Fidelity of hPolη in the presence of magnesium or manganese. (**A**) To examine the fidelity of DNA synthesis, reactions were run with 3 nM hPolη, 20 nM DNA/DNA, 100 μM individual dNTP, and 4 mM Mg^2+^ or Mn^2+^, as indicated, for 1 min. The first templating nucleotide is shown above each panel and the correct incoming nucleotides are framed below the pictures. (**B**) RNA synthesis reactions were performed as in (**A**) except using 20 nM RNA/DNA and 2000 μM rNTP for 15 min. (**C**) Time course reactions were performed with 1 nM hPolη, 20 nM RNA/DNA, 4000 μM of individual rNTPs, and 4 mM Mn for the indicated times.

Generally, Mn^2+^ is regarded as a mutagenic metal cofactor that severely reduces the fidelity of DNA polymerases. Therefore, we tested the misinsertion ability of hPolη during DNA and RNA synthesis with Mg^2+^ and with Mn^2+^. Indeed, during DNA synthesis Mn^2+^ reduced the base selectivity of the polymerase, leading to misinsertion of all three incorrect dNMPs beside the correct one, whereas Mg^2+^ supported the insertion only of the correct incoming dNMP under the conditions of the reactions (Figure 5A). In sharp contrast, during RNA extension, hPolη inserted only the correct incoming rNMP, both with Mg^2+^ and Mn^2+^ (Figure 5B). Significant incorporation of the incorrect rNMPs could not be observed even after extra-long, up to 2 h, incubation time using Mn^2+^ (Figure 5C). Still, UMP was misinserted with the highest efficiency, making it possible to determine the kinetic parameters. In Table 1, we compared the efficiency of correct rNMP insertion to UMP misinsertion opposite T, G, and C in the template. As the data show, the highest difference was opposite template G, where the correct rCMP was ~4000 times more efficiently inserted than UMP, and the lowest, ~700-fold, was detected on template T. Altogether, these results demonstrated that Mn^2+^ did not decrease the fidelity of hPolη during RNA synthesis.

### 2.4. DNA Damage Bypass during RNA Synthesis in the Presence of Manganese

**Figure 6:**
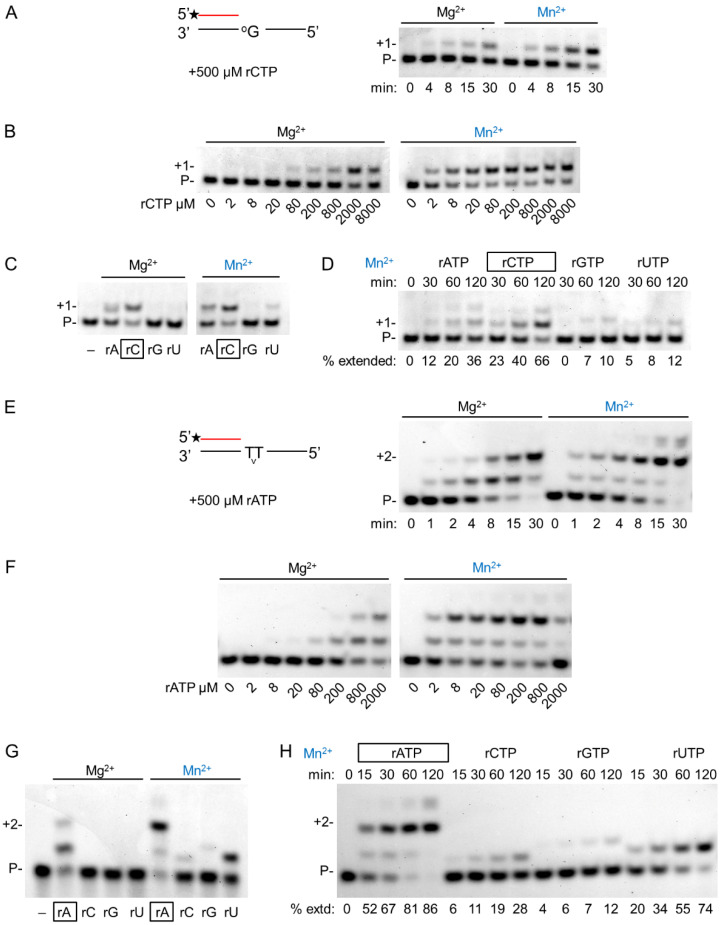
DNA damage bypass by hPolη during RNA synthesis using magnesium or manganese. (**A**–**D**) Bypass of 8-oxoG: (**A**) To check the velocity of bypass, 2 nM hPolη was incubated with 8 nM RNA/DNA and 500 μM of rCTP for the indicated times. The asterisk indicates a fluorescent label. (**B**) The affinity of hPolη to rCTP during bypass was tested using 1 nM enzyme and 8 nM RNA/DNA for 45 min with various concentrations of rCTP as indicated below each lane. (**C**) The fidelity of oxo-G bypass was examined using 8 nM RNA/DNA, 500 μM rNTP, and either 3 nM hPolη for 45 min (left panel) or 2 nM hPolη for 30 min (right panel). (**D**) A time course of misincorporation in the presence of manganese was performed with 0.8 nM hPolη, 8 nM RNA/DNA, and 4000 μM of individual rNTPs for the times indicated above each lane. In (**C**,**D**), the percentages of extended primers are shown below each lane. The correct incoming rCTP is boxed. (**E**–**H**) Bypass of TT dimer. (**E**) Bypass was assayed with 4 nM hPolη, 16 nM RNA/DNA, and 500 μM rATP for the indicated times. (**F**) Reactions contained 1 nM enzyme, 16 nM RNA/DNA, and various concentrations of rATP, as indicated below each lane. Incubation time was 45 min (**G**) Reactions were performed using 16 nM RNA/DNA, 500 μM individual rNTPs, and 3 nM hPolη for 15 min. (**H**) A time course of misincorporation in the presence of manganese was run with 1 nM hPolη, 16 nM RNA/DNA, and 500 μM of individual rNTPs for the times indicated above each lane. In (**E**–**H**) labels are the same as on (**A**–**D**).

As hPolη is a translesion synthesis polymerase, we investigated the effect of Mg^2+^ and Mn^2+^ on its DNA damage bypass ability during RNA extension opposite its two cognate DNA lesions, 8-oxoG and TT dimer. Like on undamaged templates, the velocity of insertion opposite 8-oxoG was almost the same using either Mg^2+^ or Mn^2+^ (Figure 6A and Table 1). Though the reaction was faster with Mn^2+^, the difference was less than two-fold. Likewise, near-equal velocities were observed on a TT dimer-containing template (Figure 6E). In contrast, there was a more than a hundred-fold increase in the affinity of hPolη to rNTPs in the presence of Mn^2+^ using a TT dimer or an 8-oxoG containing template resulting in an overall ~200-fold increase in efficiency (Figure 6B,F). These data indicated that the damage bypass ability of hPolη significantly improved in the presence of Mn^2+^. Though the efficiency of the enzyme was lower opposite 8-oxoG than opposite undamaged G, it was almost three times better opposite the TT dimer than on undamaged T (1.5 × 10^−1^ versus 5.2 × 10^−2^). Next, we investigated the effect of the metal cations on the base preference of hPolη during lesion bypass. As Figure 6C,D show, Mn^2+^ did not significantly alter the selectivity of the enzyme opposite 8-oxoG, since a significant insertion of rAMP and only minor rGMP and UMP insertions were observed besides the correct rCMP, using either metal ions. Surprisingly, however, considerably lower fidelity could be detected opposite a TT dimer with Mn^2+^, as evidenced by the strong misinsertion of UMP and the weak misinsertion of rCMP and rGMP besides the correct rAMP, whereas only the correct insertion of rAMP could be detected using Mg^2+^ (Figure 6G,H). 

## 3. Discussion

In this study, we investigated the metal ion dependence of the RNA synthetic activity of hPolη. We aimed to examine whether it improves by replacing the metal cofactor Mg^2+^ with other metals. The idea stemmed from our previous results showing that the weak RNA extension ability of yPolη dramatically increased when Mn^2+^ was applied instead of Mg^2+^ as a metal cofactor [29]. Mn^2+^ was previously shown to increase the activity of several other DNA polymerases like Pols β, ι, λ, µ, and PrimPol [30,31,32,33,34]. Moreover, for Polλ and Primpol, Mn^2+^ is considered an adequate metal cofactor. Mn^2+^ positively influenced both the efficiency and fidelity of these enzymes, regardless of whether dNTPs or rNTPs were used in the reactions. In sharp contrast, Mn^2+^ selectively improved the RNA synthetic activity of yPolη, whereas it compromised the DNA synthetic activity of the polymerase. 

Here we report, that similarly to its yeast counterpart, the RNA extension ability of hPolη is greatly and selectively improved in the presence of Mn^2+^. Moreover, the inability of other metal ions to support the reaction suggests that Mn^2+^ is the adequate metal cofactor of hPolη during RNA synthesis. Though Mg^2+^ also facilitates RNA extension to a small extent, it is very inefficient in agreement with published results [26]. Our experiments reveal that Mn^2+^ does not considerably alter the binding affinity of hPolη toward RNA/DNA hybrid chains or increase the velocity of the reaction, but it confers a two order of magnitude higher affinity to hPolη towards rNTPs. Importantly, in contrast to DNA synthesis, during RNA synthesis the base selectivity of hPolη is not corrupted by Mn^2+^, which is generally considered as one of the most mutagenic metals, further confirming its specific requirement for RNA extension. Moreover, Mn^2+^ improves RNA synthesis by hPolη opposite an 8-oxoG and a TT dimer as well. 

Comparing our previous and present results obtained with yeast and human Polη, respectively, we can conclude that the activities of both polymerases are similarly affected by Mn^2+^: the DNA synthetic activities of the enzymes are compromised by the severely diminished base selectivity conferred by Mn^2+^, whereas their weak RNA synthetic activities are greatly improved without affecting the fidelity. Importantly, their bypass abilities across TT dimer and 8-oxG are also significantly enhanced in the presence of Mn^2+^. However, Mn^2+^ enhances the bypass activity of yPolη several thousand-fold opposite the two lesions, as opposed to the 200-fold increase observed with hPolη. Interestingly, hPolη inserts the correct rAMP opposite a TT dimer even more efficiently than opposite an undamaged T in the presence of Mn^2+^. It suggests that TT dimer bypass is one of the main functions of hPolη, even during RNA synthesis. It is noteworthy that Mn^2+^ decreases the base selectivity of hPolη opposite the TT dimer, as we observed substantial UMP and weak rCMP and rGMP incorporations, in addition to the correct rAMP. It could indicate that hPolη needs other factors to maintain its fidelity during the bypass or that it is more substantial for the cell to accomplish the bypass and ensure the continuity of synthesis than to preserve the correct sequence in the RNA strand. Considering the second possibility, at least two cellular processes could benefit from hPolη-mediated translesion RNA synthesis: transcription and replication during Okazaki fragment synthesis. A transcriptional role of hPolη is strongly supported by our previous results indicating the involvement of yPolη in transcription elongation. By analogy with yPolη, we propose that hPolη contributes to DNA lesion bypass during transcription through its translesion RNA synthetic activity, acquiring Mn^2+^ as a metal cofactor. In that case, the lowered bypass fidelity of TT dimers can mostly go undetected owing to the redundancy of the genetic code and the fact that minor structural perturbations often leave protein function unaffected. This model is supported by recent finding showing the requirement of hPolη for the in vivo transcriptional bypass of N2-alkyl-2′-deoxyguanosine adducts [35]. Another possibility is that during the RNA primer synthesis at Okazaki fragments, hPolη could replace hPolα to carry out lesion bypass, as suggested by others [27]. We consider this possibility less likely, as primer synthesis can be reinitiated downstream of the lesion that could be bypassed by Polη through translesion DNA synthesis during the replication of the single-stranded gap. Still, even RNA primer synthesis would not be negatively affected by the lowered TT bypass fidelity of hPolη during RNA synthesis due to the subsequent degradation of the RNA part during Okazaki fragment maturation. In conclusion, through RNA translesion synthesis, hPolη could maintain the continuity of both processes, thus avoiding the severe consequences that stalled replication and transcription complexes can bring about. 

In summary, based on our results, we suggest a role for hPolη in translesion RNA synthesis during transcription. The presented data provide additional supporting evidence to our previous model, assuming that selective metal ion binding is a new regulatory mechanism contributing to the switch between the DNA and RNA synthetic activities of some polymerases. So far, this group only includes yeast and human Polη; therefore, more studies are needed to elucidate its relevance to other enzymes.

## 4. Materials and Methods

### 4.1. Protein Purification

Human Polη was overexpressed as N-terminal fusion with glutathione S-transferase (GST) in *Saccharomyces cerevisiae* BJ5464 protease deficient strain, and affinity purified on glutathione–Sepharose 4B beads (GE Healthcare, Uppsala, Sweden) using the same protocol as for yeast Polη [28]. The GST-tag was removed in the last step of the purification by incubating the beads with PreScission protease (Merck KGaA, Darmstadt, Germany). The efficiency of the purification was verified by polyacrylamide gel electrophoresis and Coomassie staining (Merck KGaA, Darmstadt, Germany), and the protein concentration was determined using a Nanodrop spectrophotometer and a gel-based assay.

### 4.2. Oligonucleotides 

Sequences of DNA/DNA and RNA/DNA primer/template substrates used for the primer extension reactions have been described previously [29]. For EMSA, Og944 5′ TTTTTTTTTTCGAGCAACTCTTGAGGCAGGCTAGGTAGCG as a template and Og530 5′ Cy3-CGCTACCTAGCCTGCCTCAAGAGTTGCTCG as DNA primer or Og531 5′ Cy3-CGCUACCUAGCCUGCCUCAAGAGUUGCUCG as RNA primer were annealed. Oligonucleotides used as primers contained a fluorophore indocarbocyanine (Cy3) label at the 5′-ends. Oligonucleotides were purchased from Integrated DNA Technologies, Coralville, Iowa, USA, except for the 8-oxoG-containing template, which was from Midland Certified Reagent Co., Midland, Texas, USA, and the TT dimer-containing oligonucleotide, which was from Trilink Biotechnologies, San Diego, California, USA. 

### 4.3. Electrophoretic Mobility Shift Assays

Purified hPolη (from 22 to 175 nM in 11 increments) was incubated with 10 nM Cy3-labelled DNA/DNA or RNA/DNA primer/template substrates in buffer R (25 mM Tris/HCl pH 8.0, 50 mM NaCl, 5 mM MgCl_2_/MnCl_2_, 1 mM DTT, 10% glycerol, 100 µg/mL BSA) for 30 min on ice. Samples were run on a 4% non-denaturing polyacrylamide gel in 0.5% TB buffer (45 mM Tris/HCl pH: 8.0) and imaged by Typhoon Trio Phosphorimager (GE Healthcare, Little Chalfont, Buckinghamshire, UK). The bound fraction was calculated using ImageQuant TL software (version 7.0, GE Healthcare, Little Chalfont, Buckinghamshire, UK) and the binding constants (binding maximum, B_max_; Hill coefficient, n; binding affinity, K_d_; and the standard deviation of each) were calculated using the SigmaPlot program (version 12.5 Systat Software, San Jose, CA, USA) by fitting to the Hill equation Y = B_max_ × X^n^/(K_d_^n^ + X^n^). 

### 4.4. Primer Extension Assays

Standard primer extension reactions (5 μL) contained 25 mM Tris/HCl pH 7.7, 10% glycerol, 100 μg/mL bovine serum albumin, 0.05% Tween-20, 2 mM DTT (added fresh), and the specified divalent cation as chloride salt, as well as substrate and enzyme as described in the figure legends. Reactions were initiated by the addition of the cation at the indicated concentrations, incubated at 37 °C, and quenched by the addition of 15 μL loading buffer containing 95% formamide, 18-mM EDTA, 0.025% SDS, 0.025% bromophenol blue, and 0.025% xylene cyanol. The reaction products were resolved on 10–14% polyacrylamide gels containing 7 M urea and analyzed with a Typhoon TRIO Phosphorimager (GE Healthcare, Little Chalfont, Buckinghamshire, UK). 

### 4.5. Determination of Steady-State Kinetic Parameters

Primer extension reactions were performed as described above with the following modifications. On undamaged templates, 1 nM hPolη was incubated with 20 nM of primer/template substrate in a standard buffer containing 4 mM MgCl_2_ or MnCl_2_. Reactions were initiated by adding the corresponding single rNTP, which varied from 250 to 6000 μM (final concentration), in 10 steps for Mg^2+^ or from 2 to 250 μM in 10 steps for Mn^2+^. Incubation at 37 °C proceeded for 35 min, 10 min, 50 min, or 45 min in cases of rATP, rCTP, rGTP, or UTP, respectively, in the presence of magnesium, and 25 min, 5 min, 15 min, or 10 min in cases of rATP, rCTP, rGTP, or UTP, respectively, in the presence of manganese. To quantitate the misincorporation of UTP in the presence of Mn^2+^, UTP was varied from 500 to 6000 μM and reactions proceeded for 60 min, 60 min, or 100 min in the cases of template T, template G, or template C, respectively. For kinetic analysis of TT dimer bypass, 1 nM hPolη was incubated with 16 nM substrate in a standard buffer. Reactions were initiated by adding rATP 200 to 3000 µM in the case of Mg^2+^ or 1 to 120 µM in the case of Mn^2+^ and incubated at 37 °C for 5 min for both. For kinetic analysis of 8-oxo-guanine bypass, 1 nM hPolη was incubated with 8 nM substrate in standard buffer. Reactions were initiated by adding rCTP 200 to 3000 µM in the case of Mg^2+^ or 1 to 120 µM in the case of Mn^2+^ and incubated at 37 °C for 30 min or 15 min using Mg^2+^ or Mn^2+^, respectively. 

The intensity of the gel bands corresponding to the substrate and the product was quantitated with Typhoon TRIO Phosphorimager using ImageQuant TL software, and the observed rates of nucleotide incorporation were plotted as a function of rNTP concentration. The data were fit by non-linear regression using the SigmaPlot program to the Michaelis–Menten equation describing a hyperbola, v = V_max_ × [rNTP]/(K_m_ + [rNTP]). The steady-state parameters k_cat_ and K_m_ and their standard deviations were obtained from the fit and were used to calculate the efficiency (k_cat_/K_m_) and the relative efficiency (activation by Mn^2+^ versus Mg^2+^) using the formula f_rel_ = (k_cat_/K_m_)_Mn2+_/(k_cat_/K_m_)_Mg2+_. 

## Figures and Tables

**Table 1:** Kinetic parameters of RNA extension with rNTPs by hPolη using Mg^2+^ or Mn^2+^ as cofactors.

Templating Nucleotide	Incoming Nucleotide	Cation	k_cat_ (min^−1^)	K_m_ (µM)	k_cat_/K_m_ (min^−1^µM^−1^)	Relative Efficiency ^a^	1/Relative Efficiency ^b^
T	rATP	Mn^2+^	0.30 ± 0.01	5.9 ± 0.7	5.2 × 10^−2^	-	1
	rUTP	Mn^2+^	0.048 ± 0.002	634 ± 144	7.5 × 10^−5^	-	690
G	rCTP	Mg^2+^	0.86 ± 0.05	1427 ± 202	6.0 × 10^−4^	1	-
	rCTP	Mn^2+^	1.27 ± 0.04	4.9 ± 0.6	2.6 × 10^−1^	430	1
	rUTP	Mn^2+^	0.064 ± 0.004	995 ± 226	6.5 × 10^−5^	-	3970
C	rGTP	Mg^2+^	0.34 ± 0.06	6260 ± 1564	5.5 × 10^−5^	1	-
	rGTP	Mn^2+^	0.54 ± 0.02	7.9 ± 0.9	6.9 × 10^−2^	1260	1
	rUTP	Mn^2+^	0.030 ± 0.004	1274 ± 519	2.4 × 10^−5^	-	2950
A	rUTP	Mg^2+^	0.37 ± 0.04	4820 ± 860	7.6 × 10^−5^	1	-
	rUTP	Mn^2+^	0.89 ± 0.03	15 ± 2.0	5.9 × 10^−2^	780	-
TT dimer	rATP	Mg^2+^	0.54 ± 0.04	630 ± 124	8.3 × 10^−4^	1	-
	rATP	Mn^2+^	0.54 ± 0.02	3.6 ± 0.6	1.5 × 10^−1^	180	-
8-oxoG	rCTP	Mg^2+^	0.11 ± 0.01	590 ± 123	1.8 × 10^−4^	1	-
	rCTP	Mn^2+^	0.18 ± 0.01	4.0 ± 0.5	4.6 × 10^−2^	260	-

^a^ Relative efficiency was calculated using the following equation: f_rel_ = (k_cat_/K_m_)_Mn2+_/(k_cat_/K_m_)_Mg2+_. ^b^ 1/Relative efficiency was calculated using the following equation: f_rel_^−1^ = (k_cat_/K_m_)_correct_/(k_cat_/K_m_)_incorrect_. k_cat_ and K_m_ values were calculated using data from at least three independent experiments.
